# *In Vitro* and *In Vivo* Comparison of Changes in Antibiotics Susceptibility of *E. coli* and Chicken's Intestinal Flora after Exposure to Amoxicillin or Thymol

**DOI:** 10.1155/2020/8824008

**Published:** 2020-07-07

**Authors:** Soukayna Hriouech, Ahmed A. Akhmouch, Mariam Tanghort, Hanane Chefchaou, Aouatef Mzabi, Najat Chami, Adnane Remmal

**Affiliations:** ^1^Department of Biology, Faculty of Science Dhar El-Mahraz, University Sidi Mohammed Ben Abdellah, P.O. Box 1796, Atlas, Fez, Morocco; ^2^Industrial Laboratory of Veterinary Alternatives (LIAV LLC), Fez, Morocco

## Abstract

This study aims at verifying, *in vitro*, the extent to which the use of amoxicillin or thymol induces the selection of resistant bacteria and at evaluating *in vivo* their effects on the development of antimicrobial resistance in the intestinal flora of poultry. *E. coli* strain was subcultured on agar plates containing increasing concentrations of either amoxicillin or thymol. Thereafter, minimal inhibitory concentrations (MICs) of thymol, amoxicillin, and two other antibiotics, tylosin and colistin, were determined using the microdilution method. Groups of chicks were subjected to a 2-week regime of either amoxicillin or thymol added to their drinking water. During the treatment with either thymol or amoxicillin, the total aerobic mesophilic flora (TAMF) was counted on thymol-gradient plates or amoxicillin-gradient plates and the MICs of antibiotics and thymol for *E. coli* isolates were determined. The *in vitro* test showed that for *E. coli*, which had been serially subcultured on increasing concentrations of amoxicillin, a 32-fold increase in MIC values for amoxicillin and a 4-fold increase for colistin and tylosin were noted. However, the MIC of thymol for this strain remained constant. For the *E. coli*, which had been serially subcultured on increasing concentrations of thymol, no change in the MIC values for antibiotics and thymol was observed. The *in vivo* test confirmed the *in vitro* one. It demonstrated that exposure to amoxicillin induced a selection of antimicrobial resistance in TAMF and intestinal *E. coli*, whereas exposure to thymol did not. The results showed that the group receiving thymol had a lower consumption index compared to the other groups. This study demonstrates the feasibility of this natural product as an alternative solution to the current use of antibiotics in poultry farming.

## 1. Introduction

For decades, antibiotics have been used in poultry as growth promoters [1]. Van Boeckel et al. [2] estimate that by 2030, a total of 105,596 (±3605) tons of antibiotics will be consumed in feed animal production globally. Industry researchers assert that antimicrobial growth promoters are essential to sustaining increases in productivity and contribute to the lowering of the cost of chicken products for consumers [3]. However, the use of antibiotics as growth promoters increases the risk of the development of antimicrobial resistance [4, 5]. Such use induces the selection of multidrug-resistant bacteria, which in turn reduces the efficacy of antibiotic therapy in both animals and humans that are colonized with resistant bacteria [1, 6]. In fact, this widespread use of antimicrobials in livestock contributes to the emergence of antimicrobial-resistant bacteria and has significant public health implications: antimicrobial-resistant bacteria of animal origin can be transmitted to humans through the environment and food products (and to agricultural workers by direct contact [2]. It is for this reason that the European Union banned the use of antibiotics as growth promoters in 2006 [7, 8]. Our laboratory has demonstrated the antimicrobial activity of essential oils and their major compounds [9–12]. Among the various constituents of EOs, thymol, the major component of the essential oil of thyme and oregano, has been shown to have an antibacterial effect on several bacterial species [10, 13–15]. These results suggest that this substance could be used as an alternative to antibiotics for poultry. This study aims to verify, *in vitro* and *in vivo*, the extent to which the use of antibiotics induces the selection of resistant bacteria and to compare its effect to thymol.

## 2. Material and Methods

### 2.1. Antibacterial Agent

Thymol was purchased from Sigma-Aldrich (France). This phenolic major compound was dispersed in a 0.2% sterile agar suspension [16].

Three antibiotics were used: amoxicillin, colistin, and tylosin. They were purchased from Sigma-Aldrich (France). These antibiotics were dispersed in distilled sterile water.

### 2.2. Effects of Amoxicillin and Thymol on *In Vitro* Susceptibility of *E. coli*

In this test, *E. coli* ATCC 25922 was used. It was provided by the Laboratory of Microbiology, Faculty of Medicine and Pharmacy of Fez, Morocco.

The ability to look for antibiotic resistance was evaluated by performing serial subcultures on Mueller Hinton (MH, Biokar®) agar plates containing increasing concentrations of either amoxicillin or thymol. Agar plates were prepared containing amoxicillin in 20 mL Trypto-casein-soy agar (TSA, Biokar®) at final concentrations of 1, 1.5, 3, 8, 12, 16, and 20 *µ*g/mL. At the same time, a second set was prepared. It contained thymol at final concentrations of 100, 200, 300, 400, 600, 900, 1200, and 1800 *µ*g/mL. The strain of *E. coli* ATCC 25922 was then subcultured successively onto prepared plates.

### 2.3. Determination of MIC

The MICs were determined by microdilution assays in 96-well plates conforming to the standards of the CLSI [17]. Ten concentrations of each agent were prepared in sterile tubes. They were carried out by successive dilutions 1/2 in Mueller Hinton broth for antibiotics and in MH broth containing agar at 0.2% for thymol. 20 *μ*l of each concentration was then added to each well containing 160 *μ*l of MHB. Bacterial suspensions were prepared by taking colonies from 24 h cultures on TSA plates. The colonies were suspended in a sterile 0.9% aqueous solution of NaCl. The density was adjusted to the turbidity of a 0.5 McFarland Standard (10^8^ colony-forming unit (CFU/mL)) [9]. These suspensions were diluted in MH broth and plated in 96-well plates at a density of 5 × 10^5^ CFU/well. After the plates were incubated at 37°C for 18 h, 40 *μ*l of 0.5% triphenyl-tetrazolium chloride (TTC) was added to each well. After two hours of incubation, the MIC corresponded to the lowest concentration that does not produce a red color [9].

### 2.4. Animals and Breeding Conditions

The chicks used in this study were a day old (approximately 40 g). They were divided into groups of ten and housed in separate cages. The photoperiod was adjusted on a daily basis to 12 hours of light and 12 hours of darkness. At the beginning of the experiment, the ambient temperature was 32°C. It was reduced by 2 to 3°C each week to finally reach 23°C at the end of the experiment. Chicks were given *ad libitum* access to feed and water. They received a maize-based feed diet that was free of antibiotics and antiparasitics.

### 2.5. Treatment in Drinking Water

- Thymol is the active principle of NP® (15% of thymol), produced by the Industrial Laboratory of Veterinary Alternatives (LIAV) in Morocco. In addition to thymol, the NP contains other excipients that provide stability and solubility. In industrial poultry farms, NP® is administered orally in drinking water at a rate of 1 g/L/day from the first day to 40^th^ day of chicken's age.
- Amoxicillin: Amoxy 70®, in powder form at a concentration of 700 mg/g, was purchased from Novovet, Casablanca, Morocco. In industrial poultry farms, it is administered orally in drinking water at a rate of 60 mg/L of body weight/day from the first day to the fifth day.

The animals were randomly divided into three experimental groups of 10 chicks each:

  Group 1 (*n* = 10): animals receiving 48 mg/L of amoxicillin in drinking water
  Group 2 (*n* = 10): animals receiving 1 g/L of NP in drinking water (equivalent to 0.15 g/L of thymol)
  Group 3 (*n* = 10) control group: animals receiving drinking water

Treatment with amoxicillin and with NP lasted 15 days (from day 7 to day 21 of the chick's age). The antimicrobial resistance of TAMF was evaluated on days 7, 14, and 21 by counting on plates containing a linear gradient from peak to trough antimicrobial concentrations of either amoxicillin or thymol. The evaluation of the antimicrobial resistance in *E. coli* isolates was performed by the evaluation of the MIC values of three antibiotics (amoxicillin, colistin, and tylosin) and thymol. Once a week (on days 7, 14, and 21), 1 g of fresh feces sample from each group was collected and solubilized in 9 ml of physiological serum, and then dilutions were made.

### 2.6. Antibiotic Gradient Plates

**Figure 1:**
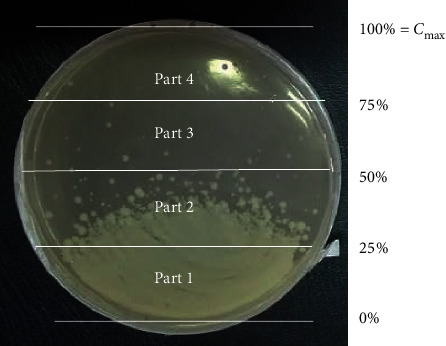
TAMF counting method on antibiotic concentration gradients.

The gradient plates were prepared as described by De Vecchi et al. [18]. Gradients were prepared in Petri dishes, on which two layers of Plate Count Agar (PCA, Biokar®) were poured. The bottom layer consisted of Plate Count Agar containing either amoxicillin or thymol at a maximum concentration (*C*_max_) allowed to harden with the plate slanted sufficiently to cover the entire bottom. The top layer, added to the dish in the normal position, did not contain any amoxicillin or thymol. For amoxicillin, three gradients were prepared from three maximum concentrations: 3, 6, and 12 *μ*g/mL. For thymol, a maximum concentration of 500 *μ*g/mL was used. 100 *μ*l of the diluted feces samples were homogeneously spread onto each plate and incubated at 37°C for 24 h. The gradient plates were analyzed by counting the colonies growing on 4 parts of each plate: from 0 to 25% *C*_max_, from 25% *C*_max_ to 50% *C*_max_, from 50% *C*_max_ to 75% *C*_max_, and from 75% *C*_max_ to *C*_max_ as shown in Figure 1.

### 2.7. Evaluation of the Antimicrobial Resistance in *E. coli*

Diluted feces samples were streaked on Eosin-Methylene Blue Agar (EMB, Biokar®). Presumptive *E. coli* colonies were identified by using Simmons' citrate and indole tests. Colonies showing negative indole results were identified by using the API 20E (bioMérieux Clinical Diagnostics, Marcy l'Étoile, France) [19]. Three *E. coli* strains were picked randomly each week. The MICs of amoxicillin, colistin, tylosin, and thymol were then determined by the microdilution method as previously described.

### 2.8. Performance Parameters

The impact of different treatments on the following zootechnical parameters was evaluated: body weight, weight gain, feed intake, and the consumption index (CI) [20].

### 2.9. Statistical Analysis

The results were expressed as mean values ± SEM (standard error of the mean). In order to compare the three groups where the independent variables were the number of TAMF, body weight, or body weight gain and the dependent variable was time, a one-way analysis of variance followed by Tukeyʼs multiple comparison test (ANOVA followed by Tukeyʼs test) was performed using Graph Pad Prism software, version 5.03. Differences of *p* < 0.05 were considered statistically significant.

## 3. Results

### 3.1. Effect of Amoxicillin on *In Vitro* Susceptibility of *E. coli* ATCC 25922 to Antibiotics

Table 1 reports the MIC values of thymol and the three antibiotics: amoxicillin, tylosin, and colistin before and after seven passages of *E. coli* ATCC25922 in the amoxicillin-containing plates. The results show that before subculture, the *E. coli* ATCC 25922 was found to be relatively sensitive to low concentrations of antibiotics. However, after seven passages in amoxicillin, a general increase in the MICs was observed for the three antibiotics. The MIC values for amoxicillin increased 32-fold. The MIC values for tylosin and colistin increased 4-fold. In the case of the thymol, no increase in the MIC was observed; it remained constant at 250 *μ*g/mL.

### 3.2. Effect of Thymol on *In Vitro* Susceptibility of *E. coli* ATCC 25922 to Antibiotics

When *E. coli* ATCC 25922 was subcultured on increasing concentrations of thymol, no growth developed on concentrations more than 300 *µ*g/mL. After the subculturing onto thymol's concentrations of 100, 200, and 300 *µ*g/mL, the evaluation of the MIC values of the three antibiotics and the thymol did not show any increase in the MIC values for neither thymol nor the three antibiotics tested. The respective MICs of amoxicillin, tylosin, and colistin were of 0.8 *μ*g/mL, 1.6 *μ*g/mL, and 0.16 *μ*g/mL. The MIC of thymol also remained constant at 250 *μ*g/mL.

### 3.3. Evaluation of the Antimicrobial Resistance to Amoxicillin in TAMF

To evaluate the effects of exposure to amoxicillin and thymol on the development of antimicrobial resistance in total aerobic mesophilic flora, groups of 10 chicks each were exposed to a 2-week course of amoxicillin or NP (thymol) added to the drinking water at a dose of 48 mg/L and 1 g/L, respectively. In the control group, chicks did not receive any form of drug. Samples of feces from different groups were taken each week (day 7, day 14, and day 21) and diluted. Then, 100 *μ*l of the diluted feces samples were spread on amoxicillin-gradient plates prepared as previously described. The total number of strains grown on amoxicillin-gradient plates is reported in Tables 2–4.

On day 7 (before treatment), and for the three groups of animals, there is a TAMF growth of 3.10^5^ CFU/g on an amoxicillin concentration less than 0.75 *μ*g/mL (Table 2). For Group 1 that received amoxicillin, after a one-week treatment (day 14), the growth of TAMF of 10^5^ CFU/g was noted on an amoxicillin concentration of 3 *μ*g/mL. On day 21, the growth was noted over the entire surface of amoxicillin-gradient plates of the three maximum concentrations tested 3 *μ*g/mL (Table 2), 6 *μ*g/mL (Table 3), and 12 *μ*g/mL (Table 4). For Group 2 treated with NP (day 7 to day 21), a TAMF growth around 10^3^ CFU g^−1^ was noted on an amoxicillin concentration less than 1.5 *μ*g/mL. As for Group 3, on days 14 and 21, bacterial growth was observed on amoxicillin concentrations less than 2.25 *μ*g/mL.

### 3.4. Evaluation of the Antimicrobial Resistance to Thymol in TAMF

The total number of strains grown on thymol-gradient plates is reported in Table 5. It shows that on day 7 (before treatment), no growth was detected on the thymol-gradient plates. On days 14 and 21, TAMF growth is noted on a thymol concentration lower than 250 *µ*g/mL. It was noted in the three groups.

### 3.5. Evolution of MIC values for *E. coli* Isolates

Table 6 reports the evolution of MIC values of thymol and the three antibiotics: amoxicillin, tylosin, and colistin during the three-week experiment. The results obtained show that before exposure to amoxicillin or thymol (day 7) and for the three groups, *E. coli* isolates had low MIC values of 0.8 *μ*g/mL for amoxicillin, 0.16 *μ*g/mL for colistin, and less than or equal to 1.6 *μ*g/mL for tylosin. The MIC values of 250 *μ*g/mL were noted for thymol. For Group 1, after a week of exposure to amoxicillin, MIC values of the *E. coli* isolates for amoxicillin had increased to 3.2 *μ*g/mL. On day 21 (after two weeks of exposure to amoxicillin), MIC values for amoxicillin had shifted to 12.8 *μ*g/mL. 16-fold increased MIC levels for colistin and tylosin during amoxicillin administration were also observed for this group. However, the MIC values for thymol did not change; it remained constant at 250 *µ*g/mL. For Group 2, after two weeks of exposure to NP, the MIC values for amoxicillin and tylosin had increased 2-fold, the MIC values for colistin had increased 4-fold while the MIC values for thymol remained stable at 250 *μ*g/mL. For Group 3, in which the chicks had received neither amoxicillin nor thymol, the MIC values of *E. coli* increased 2-fold for amoxicillin, 8-fold for colistin, and 4-fold for tylosin by day 21. The MIC values for thymol remained constant at 250 *μ*g/mL during the 3 weeks of the test.

### 3.6. Effect of Different Treatments on the Evolution of Zootechnical Parameters

#### 3.6.1. Body Weight and Body Weight Gain

**Figure 2:**
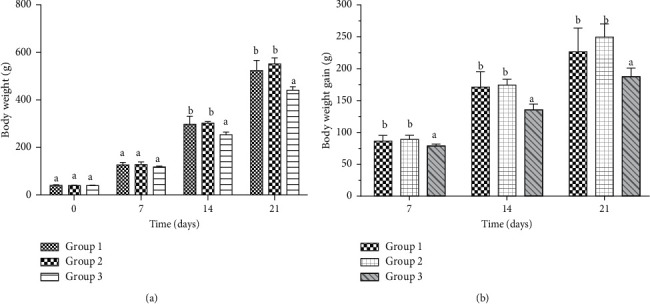
Evolution of body weight (a) and body weight gain (b). The values that include different letters are significantly different from each other at *p* < 0.05.

Figure 2 shows the evolution in time of the body weight and the body weight gain of the different groups. The results show that from the second week, the chicks' body weight of Group 1 and Group 2 began to differ significantly (*p* < 0.05) from that of the control group (Figure 2(a)). The body weight gain of the poultry in the two treated groups was found to be significantly (*p* < 0.05) higher than that of Group 3 (Figure 2(b)).

#### 3.6.2. Feed Intake and Consumption Index

The evolution in the period of time for the feed intake and the consumption index of the different groups is shown in Table 7. Throughout the experiment, the control group showed the highest consumption index.

## 4. Discussion

In the present research study, an *in vitro* assessment of the effect of amoxicillin and thymol on antimicrobial resistance in a strain of *E. coli* ATCC 25922 was performed. The effects on the antimicrobial resistance of the intestinal flora of animals *in vivo* particularly the total mesophilic aerobic bacteria and intestinal *E. coli* were evaluated.

The *in vitro* test: in order to demonstrate the effect of amoxicillin on the selection of resistance mechanisms in *E. coli* ATCC 25922, successive subcultures of this strain on increasing concentrations of amoxicillin were made. After seven subcultures, the MIC values for thymol and amoxicillin along with the antibiotics colistin and tylosin by the microdilution method were gauged. Colistin and tylosin are commonly used as feed additives in poultry production [21]. The two antibiotics colistin and tylosin were chosen to check if the phenomenon of cross-resistance was present. The results showed a 32-fold increase of the MIC values for amoxicillin. This subcultured strain is considered to be resistant to amoxicillin (MIC > 8 *μ*g/mL) according to EUCAST's recommendations [22]. These results corroborate with those obtained by Cebrian et al. [23] who showed that the *in vitro* exposure of *Salmonella* strains to amoxicillin induced a reduction in sensitivity to amoxicillin and other antibiotics. Furthermore, Qureshi et al. [24] reported similar results for *Helicobacter pylori* strains. Golikova et al. [25] also demonstrated the selection of amoxicillin-resistant *Streptococcus pneumoniae* mutants at therapeutic and subtherapeutic amoxicillin exposures in an *in vitro* dynamic model. Our results also show that *E. coli* strain became less sensitive to tylosin and colistin, while these two antibiotics were not added to the subculture medium. The MICs of tylosin and colistin increased by 4-fold compared to the starting MICs. This increase in MIC of colistin and tylosin can be explained by the existence of cross-resistance obtained between amoxicillin and the other two antibiotics. These results corroborate those obtained by Toprak et al. [26] who analyzed the evolution of resistance in *E. coli* under selection with chloramphenicol, doxycycline, and trimethoprim, and showed that after 20 days of culture with increasing concentrations of each antibiotic, the resistance levels increase dramatically. Moreover, the authors reported that the whole-genome sequencing of the evolved strains showed mutations specific to resistance to the antibiotic in use and resistance to multiple antibiotics (cross-resistance). As for the MIC of thymol, it did not change; it remained constant at 250 *μ*g/mL after subculture on increasing concentrations of amoxicillin, which leads us to consider that the selection of amoxicillin resistance does not affect the sensitivity to thymol. In order to test the *in vitro* effect of thymol on the antimicrobial resistance in the same strain of *E. coli*, successive subcultures of *E. coli* ATCC 25922 on increasing concentrations of thymol were made. During this subculture, no growth was detected on the plates containing a concentration of thymol greater than 300 *µ*g/mL. The determination of MIC values was carried out after the subculture on the three concentrations 100, 200, and 300 *µ*g/mL. The results showed that thymol does not induce the resistance selection phenomenon neither to thymol itself nor to the tested antibiotics. Several studies have reported that the use of essential oils or their major compounds does not induce the selection of resistant strains [27]. Ohno et al. [28] tested the effect of 13 essential oils on *Helicobacter pylori* strain and showed that these EOs are bactericidal against *H. pylori* without inducing the selection of resistant bacteria. Gomes Neto et al. [29] have also shown that exposure of a strain of *S. aureus* to infrainhibitory concentrations of the essential oil of *Rosmarinus officinalis* and 1,8-cineole does not induce the selection of resistant strains to these two agents.

The *in vivo* test: to confirm the *in vitro* obtained results and to verify whether the use of either amoxicillin or thymol in the drinking water of the chicks will cause a selection of resistant bacteria *in vivo*, a test was carried out by treating groups of chicks with amoxicillin or NP which contains thymol as an active principle. Then, samples of chick feces were collected to evaluate the sensitivity of total aerobic mesophilic flora and intestinal *E. coli*. The results of TAMF's counting on amoxicillin-gradient plates showed that following amoxicillin administration to the drinking water (day 7 to day 21), total aerobic mesophilic flora became less sensitive to amoxicillin; this is illustrated by the growth over the entire gradient surface of the three maximum concentrations of amoxicillin (3, 6, and 12 *µ*g/mL) by the day 21, whereas before the addition of amoxicillin to the drinking water, growth was only observed at concentrations less than 0.75 *µ*g/mL. However, for the group receiving NP, the growth of TAMF is noted just on concentrations of amoxicillin less than 1.5 *µ*g/mL. These results show that the exposure to amoxicillin exerts a selective pressure for the emergence of resistance in TAMF, whereas the addition of the NP does not induce this phenomenon. For the control group, on days 14 and 21, growth was observed on amoxicillin concentrations less than 2.25 *µ*g/mL whereas, on day 7, growth was observed on concentrations less than 0.75 *µ*g/mL. These observations suggested that environmental sources including feed, water, and air may be the main factors in the colonization of the chicks' intestinal tracts by less sensitive bacteria [30, 31]. As for the count of TAMF on thymol-gradient plates, the same results were noted for the three groups; no growth was noted on day 7, whereas growth was noted on a thymol concentration less than 250 *µ*g/mL on days 14 and 21. The absence of growth on day 7 can be explained by the low bacterial load in the feces suspension prepared on day 7 or the absence of certain bacterial strains which subsequently colonize the intestine from the feed, the water, or the air [30].

After observing the effect of NP and amoxicillin on the intestinal TAMF, it was necessary to confirm this effect on intestinal *E. coli* isolates. The results obtained show a significant increase by 16-fold in MIC values of amoxicillin for *E. coli* isolated from the group that received amoxicillin. These results are similar to those obtained by Van der Horst et al. who showed that the addition of amoxicillin, enrofloxacin, or oxytetracycline in the chicken's drinking water induced the selection for resistant *E. coli* [32]. Similarly, Miranda et al. and Simoneit et al. reported that the administration of amoxicillin or other antibiotics in drinking water induced the selection and development of antimicrobial resistance in *E. coli* strains from chickens [33, 34]. Moreover, Pouwels et al. [35] reported that amoxicillin, which is mainly prescribed for human infections, is associated with increased resistance against various antibiotics among *E. coli*. For the group that received amoxicillin, an increase by 16-fold in MICs of tylosin and colistin was also observed. This result confirms the presence of the phenomenon of cross-resistance. Concerning the control group, the MIC values for amoxicillin increased 2-fold, for tylosin 4-fold, and for colistin 8-fold by day 21, although this group did not receive any kind of drug. This can be explained by the spread of less sensitive strains from a contaminated environment. Strains of antimicrobial-resistant *E. coli* were isolated from the feed, water, and litter of chickens in India [36]. Da Costa et al. and Rossato et al. also reported that feed is a source of antimicrobial-resistant *E. coli* [37, 38]. For the group receiving NP, the MIC values noted are lower compared to those noted in the control group, and an increase in the MIC by 2-fold for amoxicillin and tylosin and 4-fold for colistin was observed. Regarding the MIC of thymol, it was 250 *µ*g/mL for the three groups. This confirms the results obtained *in vitro* which show the absence of development of thymol resistance.

To evaluate the effect of NP on the zootechnical performances of animals, we evaluated the body weight, the weight gain, the feed consumption, and the consumption index. The results obtained showed that the weight of the treated groups was significantly higher than that of the control group and that group receiving NP had the lowest consumption index. This can be explained by the reduction of the bacterial load by thymol, which affects intestinal integrity [39]. With a balanced intestinal flora, food may be more easily absorbed, which explains the difference in growth between animals. A study conducted by Lee et al. showed that thymol increases the activity of chicken's intestinal amylase which improves chicken growth by increasing the digestibility of nutrients and the regulation of the intestinal microflora [40]. Ezzak Abd El-Hack et al. [41] also demonstrated the valuable potential of thymol to enhance the growth performances, digestive enzyme activity, nutrient bioavailability, immunity, and general health of poultry. Suresh et al. [1] have also recommended the use of essential oils and their compounds as alternatives to antibiotics as it could minimize the risk of antibiotic resistance in livestock. Therefore, thymol could represent a natural alternative to antibiotics currently used in poultry farming as reported by Yang et al. [42].

## 5. Conclusion

The results of these experiments lead us to conclude that thymol does not induce the selection of antimicrobial-resistant bacteria. However, it has a significantly positive effect on the zootechnical performance of the animals, making it a good, safe alternative that meets the breeders. This natural product could be an efficient alternative that would have positive effects on the fight for antimicrobial resistance that is observed in human medicine and that originates from the administering of antibiotics to farm animals.

## Figures and Tables

**Table 1:** Summary of *E. coli* MICs of antibiotics and thymol.

	MICs (*µ*g/mL)	
	Starting MICs	After seven subcultures on amoxicillin
Amoxicillin	0.8	25.6
Colistin	0.16	0.64
Tylosin	1.6	6.4
Thymol	250	250

**Table 2:** Number of total aerobic mesophilic flora on the amoxicillin-gradient plate with *C*_max_ = 3 *µ*g/mL.

	Total aerobic mesophilic flora in log_10_ CFU/g											
	Day 7 (before treatment)				Day 14				Day 21			
Amoxicillin-gradient (*µ*g/mL)	0–0.75	0.75–1.5	1.5–2.25	2.25–3	0–0.75	0.75–1.5	1.5–2.25	2.25–3	0–0.75	0.75–1.5	1.5–2.25	2.25–3
Group 1	>5.48 ± 00	0	0	0	>5.48 ± 00b	>5.48 ± 00c	>5.48 ± 00c	4.34 ± 0.02b	>5.48 ± 00c	>5.48 ± 00c	>5.48 ± 00c	>5.48 ± 00
Group 2	>5.48 ± 00	0	0	0	3.81 ± 0.03a	3.38 ± 0.09a	0a	0a	4.11 ± 0.03a	3.92 ± 0.03a	0a	0a
Group 3	>5.48 ± 00	0	0	0	3.73 ± 0.04a	3.65 ± 0.03b	3.65 ± 0.04b	0a	4.16 ± 0.01b	4.08 ± 0.002b	3.95 ± 0.04b	0a

The values that include different letters are significantly different from each other at *p* < 0.05.

**Table 3:** Number of total aerobic mesophilic flora on the amoxicillin-gradient plate with *C*_max_ = 6 *µ*g/mL.

	Total aerobic mesophilic flora in log_10_ CFU/g											
	Day 7 (before treatment)				Day 14				Day 21			
Amoxicillin-gradient (*µ*g/mL)	0–1.5	1.5–3	3–4.5	4.5–6	0–1.5	1.5–3	3–4.5	4.5–6	0–1.5	1.5–3	3–4.5	4.5–6
Group 1	0	0	0	0	>5.48 ± 00c	4.46 ± 00c	0	0	>5.48 ± 00c	>5.48 ± 00c	>5.48 ± 00	>5.48 ± 00
Group 2	0	0	0	0	3.38 ± 0.08a	0a	0	0	4.26 ± 0.11a	0a	0	0
Group 3	0	0	0	0	4.59 ± 0.005b	3.84 ± 0.06b	0	0	4.87 ± 0.015b	4.11 ± 0.002b	0	0

The values that include different letters are significantly different from each other at *p* < 0.05.

**Table 4:** Number of total aerobic mesophilic flora on the amoxicillin-gradient plate with *C*_max_ = 12 *µ*g/mL.

	Total aerobic mesophilic flora in log_10_ CFU/g											
	Day 7 (before treatment)				Day 14				Day 21			
Amoxicillin-gradient (*µ*g/mL)	0–3	3–6	6–9	9–12	0–3	3–6	6–9	9–12	0–3	3–6	6–9	9–12
Group 1	0	0	0	0	5.43 ± 0.05c	0	0	0	>5.48 ± 00c	>5.48 ± 00	>5.48 ± 00	>5.48 ± 00
Group 2	0	0	0	0	3.50 ± 0.20a	0	0	0	4.34 ± 0.02a	0	0	0
Group 3	0	0	0	0	4.75 ± 0.04b	0	0	0	4.93 ± 0.02b	0	0	0

The values that include different letters are significantly different from each other at *p* < 0.05.

**Table 5:** Number of total aerobic mesophilic flora on the thymol-gradient plate with *C*_max_ = 500 *µ*g/mL.

	Total aerobic mesophilic flora in log_10_ CFU/g											
	Day 7 (before treatment)				Day 14				Day 21			
Thymol-gradient (µg/mL)	0–120	120–250	250–370	370–500	0–120	120–250	250–370	370–500	0–120	120–250	250–370	370–500
Group 1	0	0	0	0	4.68 ± 0.02	4.06 ± 0.02	0	0	4.78 ± 0.01	4.21 ± 0.07	0	0
Group 2	0	0	0	0	4.67 ± 0.01	4.09 ± 0.05	0	0	4.81 ± 0.03	4.20 ± 0.03	0	0
Group 3	0	0	0	0	4.68 ± 0.02	4.05 ± 0.06	0	0	4.81 ± 0.02	4.21 ± 0.04	0	0

**Table 6:** Summary of antibiotics and thymol minimal inhibitory concentration (MIC) values in *E. coli* isolates from feces sample.

		MIC in *μ*g/mL		
		Day 7 (before treatment)	Day 14	Day 21
Group 1 (Amox)	Amoxicillin	0.8	3.2	12.8
	Colistin	0.16	1.28	2.56
	Tylosin	1.6	6.4	25.6
	Thymol	250	250	250

Group 2 (NP)	Amoxicillin	0.8	1.6	1.6
	Colistin	0.16	0.16	0.64
	Tylosin	0.8	0.8	1.6
	Thymol	250	250	250

Group 3 (control)	Amoxicillin	0.8	0.8	1.6
	Colistin	0.16	0.64	1.28
	Tylosin	0.8	1.6	3.2
	Thymol	250	250	250

**Table 7:** Evolution of the feed intake and consumption index.

	Feed intake (g)			Total feed intake	Consumption index (CI)			Global CI
Groups	Day 0–day 7	Day 7–day14	Day 14–day 21		Day 0–day 7	Day 7–day14	Day 14–day 21	
Group 1 (Amox)	322	605	677	1604	3,73	3.54	2.99	3.31
Group 2 (NP)	308	533	578	1419	3.46	3.05	2.3	2.76
Group 3 (control)	350	616	820	1786	4.45	4.54	4.38	4.45

## Data Availability

The data used to support the findings of this study are available from the corresponding author upon request.
